# Fractional Young double-slit numerical experiment with Gaussian wavepackets

**DOI:** 10.1038/s41598-020-76512-5

**Published:** 2020-11-10

**Authors:** Mahboubeh Ghalandari, M. Solaimani

**Affiliations:** Department of Physics, Qom University of Technology, Qom, Iran

**Keywords:** Applied optics, Optical physics

## Abstract

In the present work, we consider the transmission properties of a Gaussian wavepacket when transmits through few double and multi-slit systems in a fractional medium. For this purpose, we have solved the two-dimensional fractional Schrodinger equation utilizing a split-step Fourier method. Then, we have investigated the effects of different parameters such as the number of slits, slit width, barrier width, layer width, layer heights, fractional order, and wavepacket width on the transmission coefficient, and wavepacket evolution.

## Introduction

Young double-slit experiment (the heart of quantum physics as regarded by Richard Feynman^1^ that is impossible, absolutely impossible, to explain in any classical way) is a famous interference one that is usually used to show the wave properties of particles. The quantum mechanical description of the double-slit experiment can change one's ideas about different classical concepts such as waves, particles, movement, location, etc. In a double-slit experiment on 1909 by Taylor, a very dim light emits just a single photon at a time, led to Paul Dirac’s famous claim: each photon interferes only with itself^2^.

Within a double-slit experiment, a monochromatic source S propagates light that passes through two slits S_1_ and S_2_. These slits are then two sources of coherent light. Therefore, if these two slits have the same sizes, then the light waves emitted from these slits have the same amplitudes. This experiment leads to a fringe pattern that the fringes separation is inversely proportional to the slit separation. Slit diffraction has vast applications in holography, acousto-optics, spectroscopy, etc.^3–5^. These applications lead to the attraction of great interest to this field of study.

So far, in addition to fabrication double-slit-grating^6^, others fields such as double-slit like interference by a pseudocereal metamaterial slab^7^, the double-slit experiment of surface Plasmon polaritons excited by mercury-lamp light source^8^, laser^9,10^, and single photons^11^, have been investigated. Besides, Young’s double nanoslit study with plasmon hybridization^12^, Plasmonic wave plate based on subwavelength nanoslits^13^, periodic metallic nano-slits^14^, three-dimensional quantum slit diffraction^15^, enhanced optical transmission of non-coaxial double-layer nano-slit with slanted sidewall arrays^16^, double-slit phenomena by using near field imaging technique^10,14^, off-centered double-slit metamaterial for elastic wave polarization anomaly^17^, interference of surface waves in a metallic nanoslit^18^, etc. have thus far been considered. In double-slit diffraction experiments, loss of coherence^19^, parameter estimation by decoherence^20^, squeezing and slowed quantum decoherence^21^, etc. are also investigated. From a geometric standpoint, the double-slits are the basic components of multi-slits. In this way, extraordinary optical transmission comprehension of 1D period nano-slits arrays^22^ has been reported.

In the literature, diffraction experiments on the forming Young’s double-slit fringes pattern by individual particles^23–25^ and single wave-driven particles^26^ have also been reported. For example, double-slit experiments with electron^27,28^, neutrons, atoms, molecules^29–33^, small clusters^34^, and even large molecules like C_60_^35^, have till now been considered. In addition to particles, the double-slit experiment with wavepackets is also possible. The double-slit experiment with Gaussian wave packets can include the three cases of plane waves, wave packets narrower than the slit size, and even the intermediate situations. One and two slits studies by Gaussian wave packets in the presence or absence of the interactions with the wall have previously been studied^36–38^. Therefore, along with above-mentioned particles, diffraction of Gaussian wave packets by a single slit^39^, effects of gravity and nonlinearity on dynamics of macroscopic wave packet passing through double-slits^40^, studying the two-dimensional electron wave packet passing through a double-slit by finite difference solving of the time-dependent Schrodinger equation^41^, near and intermediate fields of an ultra-short pulse transmitted through Young’s double-slit experiment^42^, have also been considered.

Leibniz proposed the generalization of differentiation to fractional order^43^. Thereafter, the fractional calculus found different applications, including turbulence, complex networks, dielectric relaxations, phase transition, visco-elastic materials, control systems, etc.^44^. Longhi^45^ proposed an application of the fractional Schrödinger equation to the optics. In optics, the fractional Laplacian means a non-parabolic dispersion, i.e., the dispersion of the system directly changes. Although, in comparison to the standard Schrodinger equation, the fractional one just contains the fractional Laplacian operator instead of the common one, this change can lead to significant differences in the wave function characteristics. In the space-fractional Schrödinger formalism, optical solitons, self-focusing, and wave collapse^46^, Hermite–Gaussian-like solitons^47^, solitons in a 1D array of rectangular ferroelectric nanoparticles^48^, nontrivial wave-packet collision and broadening^49^, parity-time-symmetric lattice potentials^50^, defect modes^51^, modulation instability of Co-propagating optical beams^52^, propagation characteristics of ring Airy beams^53^, transmission through locally periodic potentials^54^, localization and Anderson delocalization of light^55^, quantum information entropies^56^, etc. have thus far been studied.

In this work, we have considered the fractional Young double-slit experiment with incident Gaussian wavepackets in numerical treatment. We have solved the time-dependent nonlinear fractional Schrodinger equation by using a split step Fourier method. Then, we have studied the transmission of a Gaussian wavepacket through double and multi-slits as well as the wavepacket evolution in the fractional calculus formalism. In the available literature on the double slit studies, the researchers mainly investigated the diffraction pattern. However, there are a number of papers that they also studied the transmission effect^16,22,54^. In our present study, in addition to the diffraction, we have also studied the transmission properties of multi-slit systems in the conventional and fractional mediums.

## Formalism

The wavepacket propagation in the two-dimensional fractional Schrodinger equation formalism can be studied by using:

1 $$ i\frac{\partial \psi}{\partial t}  = \left[ {{\varvec{\upbeta}} \text Q_\text{R} \left(\mathrm{{x,y,} {\textit t},\alpha} \right) \left( { - \Delta ^{2}} \right)^\frac{\alpha} {2} - \gamma \left| {\psi \left({x,y,t} \right)} \right|^{2} + \text M\left({\mathrm{x,y,}} {p_x,p_y,{\textit t},\alpha} \right)} \right]\psi \left( {x,y,t} \right) $$

where $\alpha$, $\varvec{\upbeta}$, and $\gamma$ are the fractional derivative order, Laplacian coefficient, and nonlinear interaction strength, respectively. We also have $\Delta^{2} = {{\partial^{2} } \mathord{\left/ {\vphantom {{\partial^{2} } {\partial x^{2} }}} \right. \kern-\nulldelimiterspace} {\partial x^{2} }} + {{\partial^{2} } \mathord{\left/ {\vphantom {{\partial^{2} } {\partial y^{2} }}} \right. \kern-\nulldelimiterspace} {\partial y^{2} }}$. Also, Q_R_ and M are real and complex functions, respectively. Let us assume that

2 $$\text M \left({\mathrm{x,y,}} {p_x,p_y,{\textit t},\alpha} \right) = \text{V}\left( {x,y} \right) + i\varvec{\upbeta} \text{Q}_\text{I} \left(\mathrm{{x,y}, {\textit t},{\alpha}} \right)\left( { - \Delta ^{2}} \right)^\frac{\alpha} {2}$$

where the geometrical potential $\text V\left( {x,y} \right)$ is defined for the double slit problem, and $\varvec{\upbeta} \text Q_\text I \left( \mathrm{{x,y,}{ \textit t,\alpha}} \right)$ is a real function that determines amplitude of imaginary part of the potential. By substituting Eq. (2) in Eq. (1), we have

3 $$ i\frac{\partial \psi}{\partial t}  = \left[ {{\varvec{\upbeta}} \text Q \left(\mathrm{{x,y,} {\textit t},{\alpha}} \right) \left( { - \Delta ^{2}} \right)^\frac{\alpha} {2} - \gamma \left| {\psi \left({x,y,t} \right)} \right|^{2} + \text{V}\left(\mathrm {x,y} \right)} \right]\psi \left( {x,y,t} \right) $$

where $$ \text Q \left( \mathrm{{x,y,} {\textit t},\alpha} \right) = \text Q_R \left( \mathrm{{x,y,}{\textit t},\alpha} \right) +\text {iQ}_\text I \left(\mathrm{ {x,y,}{\textit t},\alpha} \right) $$.

Note that when $\text Q \left(\mathrm{{x,y,} {\textit t},\alpha} \right) =  \exp \left( 2 \pi i \right) = 1$, we obtain the usual NFSE. In this paper however, we investigated a more general case of $\text Q \left( \mathrm{{x,y,}{\textit t},\alpha} \right)$ as follows

4 $$  \text Q \left( \mathrm{{x,y,} {\textit t},\alpha} \right) = \exp \left[ \frac{i \pi \alpha}{2 \left| g \left(\mathrm{ {x,y,} {\textit t}} \right) \right| } \left( \left| g \left( \mathrm{{x,y,} \textit t} \right) \right| - g \left( \mathrm{{x,y},{\textit t}}\right) \right) \right]$$

where $g \left(\mathrm {x,y,} {\textit t}\right) = -i \frac {\Delta \psi \left( {x,y,t} \right) }{\psi \left( {x,y,t} \right) }$ is a real function. The fractional derivative for the fractional parameter (Lévy index) $1 < \alpha \le 2$ can be defined as^57^,

5 $$ \frac{{\partial^{\alpha } }}{{\partial \left| x \right|^{\alpha } }}\psi \left( {x,t} \right) = \frac{1}{2\cos (\alpha \pi /2)\Gamma (2 - \alpha )}\frac{{d^{2} }}{{dx^{2} }}\int\limits_{ - \infty }^{\infty } {\left| {x - \xi } \right|^{1 - \alpha } \psi (\xi ,t)d\xi } , $$

where $\Gamma$ is the gamma function Also, the term $V\left( {x,y} \right)$ is the geometrical potential that we have defined it for a double-slit problem as,

6 $$ V(x,y) = \left\{ {\begin{array}{*{20}l} 0 \hfill & {\quad All\;x;} \hfill & {\quad y < y_{1} } \hfill \\ {V_{0} } \hfill & {\quad x < x_{1} ;} \hfill & {\quad y_{1} < y < y_{2} } \hfill \\ 0 \hfill & {\quad x_{1} < x < x_{2} ;} \hfill & {\quad y_{1} < y < y_{2} } \hfill \\ {V_{0} } \hfill & {\quad x_{2} < x < x_{3;} } \hfill & {\quad y_{1} < y < y_{2} } \hfill \\ 0 \hfill & {\quad x_{3} < x < x_{4;} } \hfill & {\quad y_{1} < y < y_{2} } \hfill \\ {V_{0} } \hfill & {\quad x_{4} < x;} \hfill & {\quad y_{1} < y < y_{2} } \hfill \\ 0 \hfill & {\quad All\;x;} \hfill & {\quad y > y_{2} } \hfill \\ \end{array} } \right. $$

**Figure 1:**
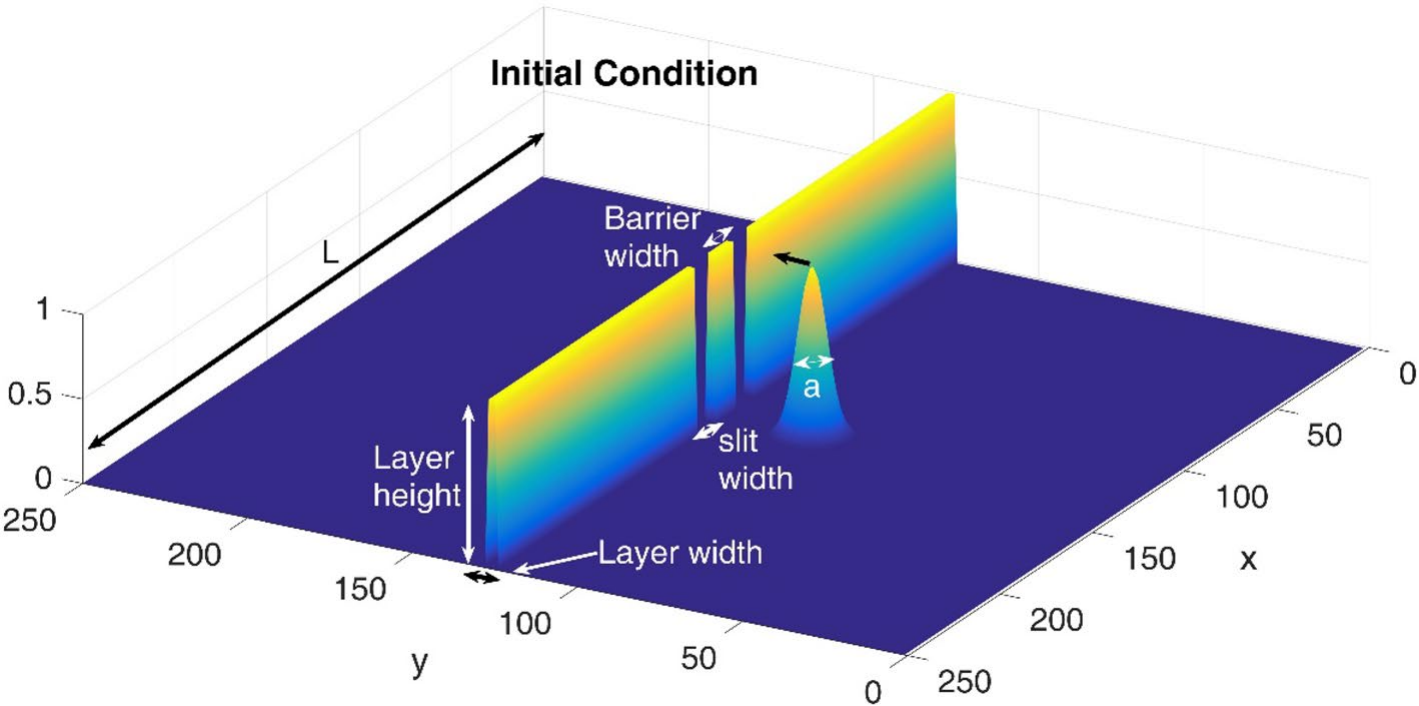
A schematic illustration of the initial condition including the Gaussian wavepacket and the double-slit setup.

Also, for a multi-slit problem we use the constant total length effective potential as^58^,

7 $$ V(x,y) = \left\{ {\begin{array}{*{20}c} {V_{0} } & {i = 1,3, \ldots } & {\frac{i - 1}{{2N + 1}}L < x < \frac{i}{2N + 1}L;} & {y_{1} < y < y_{2} } \\ 0 & {i = 2,4,i = 2,4,} & {\frac{i - 1}{{2N + 1}}L < x < \frac{i}{2N + 1}L;} & {y_{1} < y < y_{2} } \\ 0 & {} & {All\;x;} & {y < y_{1} } \\ 0 & {} & {All\;x;} & {y > y_{2} } \\ \end{array} } \right. $$

where *N*, *V*_0_, and L are the number of wells, the potential height, and the system length, respectively. Also, i shows the i’th well or barrier. The potential term is the rigid-body potential which describes the slit wall^59^. In the following, we assume the following initial Gaussian wave packet at $t = 0$,

8 $$ \psi \left( {x,y,t = 0} \right) \equiv \exp \left[ { - \frac{{(x - x_{0} )^{2} + (y - y_{0} )^{2} }}{{2a^{2} }} + iky} \right] $$

where it represents a traveling Gaussian wave-packet. Here, ($x_{0}$, $y_{0}$(, $a$ and $k$ indicate the center of the wave packet, width of the wave packet and wave packet wave vector, respectively. A schematic illustration of the initial condition, including the Gaussian wavepacket and the double-slit setup, is presented in Fig. 1. We use a split step Fourier method^60^ to solve the Eq. (1) and to study the wave-packet evolution. Then, we use the following relations for reflection ($R$) and transmission coefficients ($T$). We calculated these quantities at an enough long time after the collision of the wave packet on the double or multi-slit system^61,62^,

$$ R = \int\limits_{ - \infty }^{ + \infty } {\int\limits_{ - \infty }^{{y_{1} }} {\left| {\psi (x,y,t)} \right|^{2} } dydx} $$

$$ T = \int\limits_{ - \infty }^{ + \infty } {\int\limits_{{y_{2} }}^{ + \infty } {\left| {\psi (x,y,t)} \right|^{2} } dydx} $$

where

$$ R + T = 1 $$

## Results and discussions

In the current paper, we have simulated the double and multi-slits experiment in the fractional two dimensional Schrodinger equation formalism. We have used a split step Fourier method to solve the corresponding Schrodinger equation numerically. Then, by using a few integrations, we have evaluated the transmission coefficient when a Gaussian initial wavepacket impinges on the mentioned double and multi-slits systems. Thereafter, we have tried to evaluate the effects of different parameters such as slit width, slit height, wavepacket width, etc. on the wavepacket evolution and transmission characteristics. These geometrical parameters have also been illustrated in Fig. 1.

**Figure 2:**
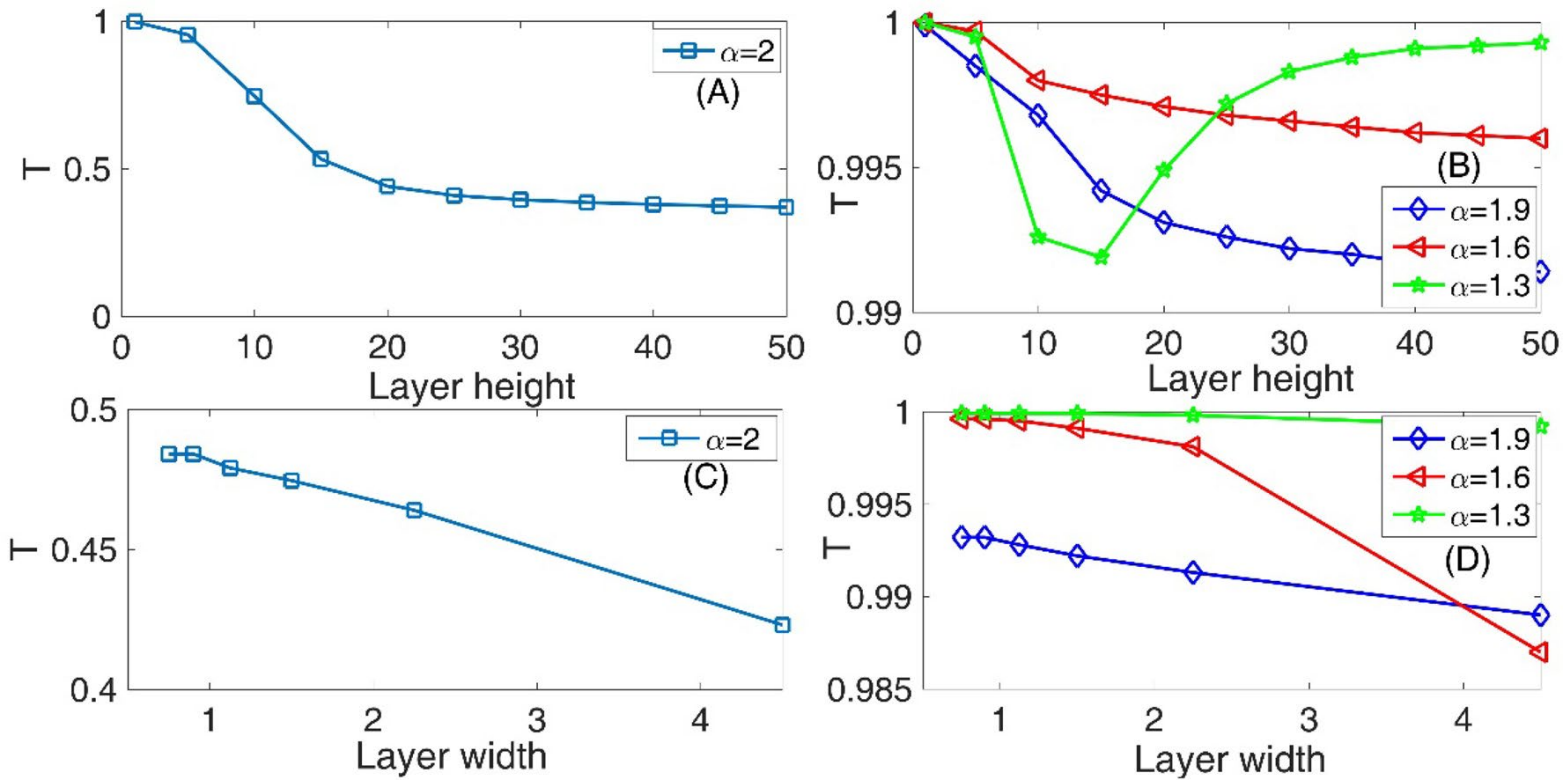
Panel (**A**) Variation of the transmission coefficient T as a function of the layer height for standard non-fractional Schrodinger equation with $\alpha = 2$ and layer width = L/30. Panel (**B**) The same as the panel (**A**) but for three fractional orders *α* = 1.9, 1.6, and 1.3. Panel (**C**) Variation of the transmission coefficient T as a function of the layer width for standard non-fractional Schrodinger equation with $\alpha = 2$. Panel (**D**) The same as the panel (**C**) but for three fractional orders α = 1.9, 1.6, and 1.3. In this figure, we assumed system length L = 45, initial Gaussian wave parameter ‘a’ = 0.7, number of slits = 2, slit width = L/100, barrier width = L/60, Laplacian coefficient *β* = 0.5, and nonlinearity strength *γ* = 0.

In the panel (A) of the Fig. 2, we have presented the variation of the transmission coefficient T as a function of the layer height for standard non-fractional Schrodinger equation with $\alpha = 2$ and layer width = L/30. Panel (B) of this figure is also the same as the panel (A) but for three fractional orders *α* = 1.9, 1.6, and 1.3. In this figure, we assumed system length L = 45, initial Gaussian wave parameter a = 0.7, the number of slits = 2, the slit width = L/100, the barrier width = L/60, the Laplacian coefficient *β* = 0.5, and the nonlinearity strength *γ* = 0. As this figure shows, by increasing the barrier height, the transmission coefficient decreases to the standard Schrodinger equation in the panel (A). However, this fact is consistent with our common physics insight. In this figure, increasing the layer height to 50 can decreases the transmission coefficient by 50%. Using a small decrease of the fractional parameter $\alpha$ to 1.9 in the panel (B), we see that, the transmission coefficient again decreases when the layer height increases. But in this case, the amount of decreases in the transmission coefficient is very small compared to the standard Schrodinger equation in the panel (A). Also, by decreasing the fractional parameter $\alpha$, we see that the transmission coefficient increases. In a strongly fractional Schrodinger equation with *α* = 1.3, the transmission coefficient at first decreases and then increases if layer height increases. This fact is not consistent with our common physical insight. Panel (C) of this figure shows the variation of the transmission coefficient T as a function of the layer width for standard non-fractional Schrodinger equation with $\alpha = 2$. Also, panel (D) is the same as the panel (C) but for three fractional orders *α* = 1.9, 1.6, and 1.3. In these two panels, we see that by increasing the layer width the transmission coefficient decreases. Also, by increasing the fractional parameter $\alpha$, the transmission coefficient increases. In the strongly fractional systems with *α* = 1.3, the layer width is a less critical parameter in the transmission coefficient because in all of the studied layer widths, the transmission coefficient is approximately 100%.

**Figure 3:**
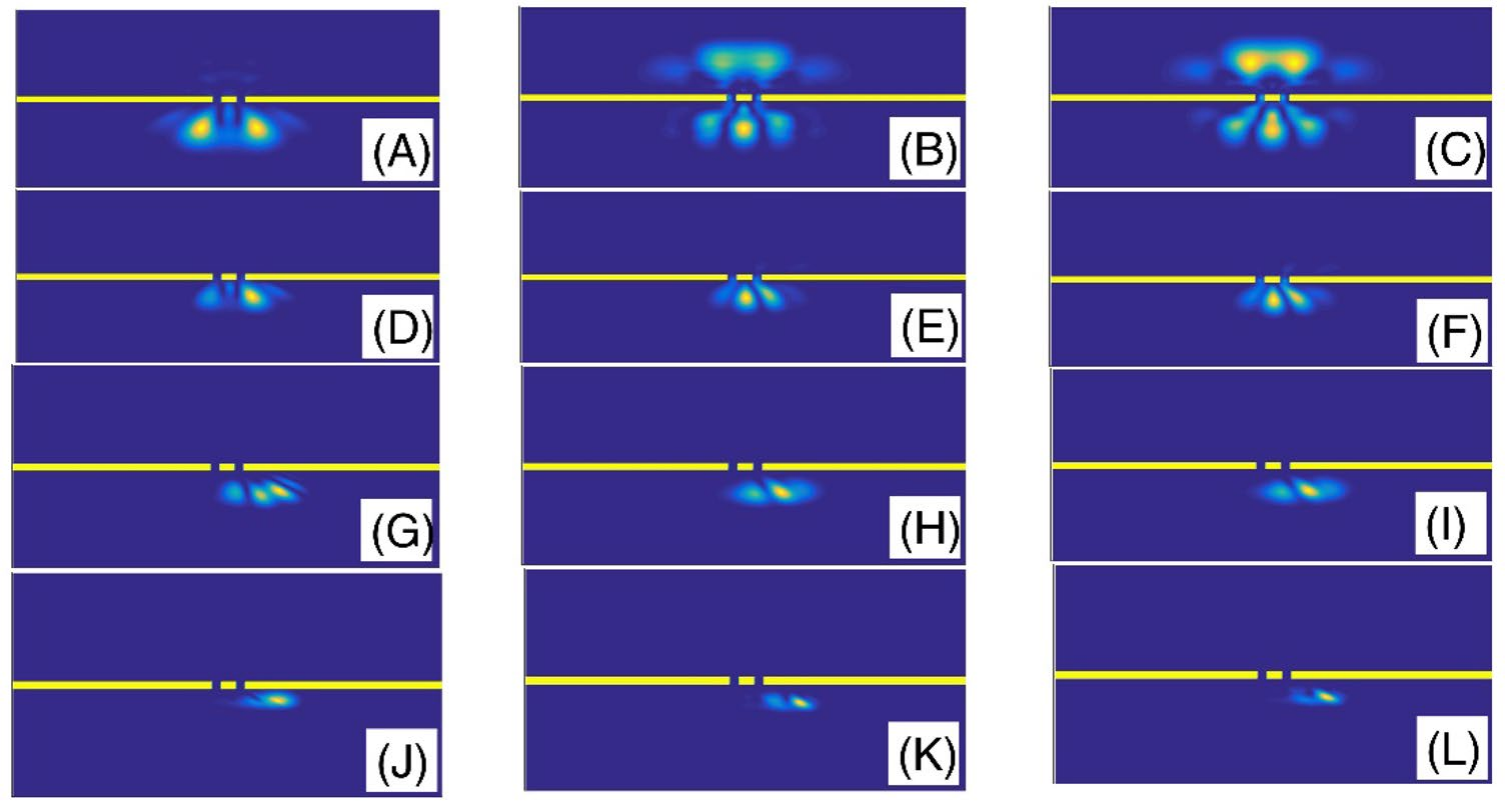
The final shape of the Gaussian wavepacket at sufficiently large time in the panels (**A**–**L**) are plotted for (*α*, layer height) = (2, 5), (2, 25), (2, 50), (1.9, 5), (1.9, 25), (1.9, 50), (1.6, 5), (1.6, 25), (1.6, 50), (1.3, 5), (1.3, 25), and (1.3, 50), respectively. Other parameters are the same as the panels (**A**) of the Fig. 2. In each panel, the wavepacket has moved downward.

**Figure 4:**
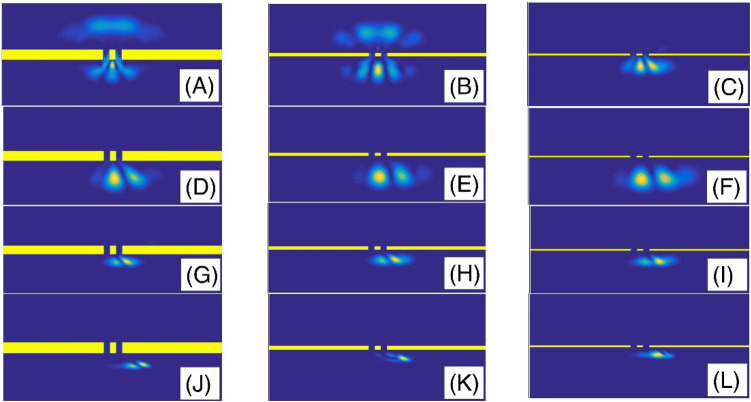
The final shape of the Gaussian wavepacket at sufficiently large time in the panels (**A**–**L**) are plotted for (layer width,*α*) = (L/10, 2), (L/30, 2), (L/60, 2), (L/10, 1.9 ), (L/30, 1.9), (L/60, 1.9), (L/10, 1.6), (L/30, 1.6), (L/60, 1.6), (L/10, 1.3), (L/30, 1.3), and (L/60, 1.3), respectively. Other parameters are the same as the panels (**C**) of the Fig. 2. In each panel, the wavepacket has moved downward.

In the Fig. 3, we have presented the final shape of the Gaussian wavepacket after a sufficiently large evolution time. Panels (A) to (L) are plotted for ($\alpha$, layer height) = (2, 5), (2, 25), (2, 50), (1.9, 5), (1.9, 25), (1.9, 50), (1.6, 5), (1.6, 25), (1.6, 50), (1.3, 5), (1.3, 25), and (1.3, 50), respectively. Other parameters are the same as the panels (A) of the Fig. 2. In each panel, the wavepacket has moved downward. Here, the simulation of double-slit experiment shows a few fringes. By passing the wavepacket through the double-slit system, some parts will split into two sections at the transmission region. Therefore, each slit behaves like a light-emitting source. Here, the diffracted wave packet in the Young double-slits experiment indicates the interferences with few visible peaks. The progression to a smaller fractional parameter $\alpha$, generally speaking, shows a pattern of narrowing the intensity peaks. The pattern includes a series of dark and bright fringes. In a bright fringe, the constructive interference occurs while in a dark fringe, destructive interference occurs. In these panels, it is clear that, by increasing the layer height, the transmission coefficient decreases. Figure 4 also shows the final shape of the Gaussian wavepacket at a sufficiently large evolution time. The panels (A) to (L) are plotted for (layer width,$\alpha$) = (L/10, 2), (L/30, 2), (L/60, 2), (L/10, 1.9 ), (L/30, 1.9), (L/60, 1.9), (L/10, 1.6), (L/30, 1.6), (L/60, 1.6), (L/10, 1.3), (L/30, 1.3), and (L/60, 1.3), respectively. Other parameters are the same as the panels (C) of the Fig. 2. In each panel, the wavepacket has moved downward. As these panels also show, by decreasing the barrier width, the transmission coefficient increases.

**Figure 5:**
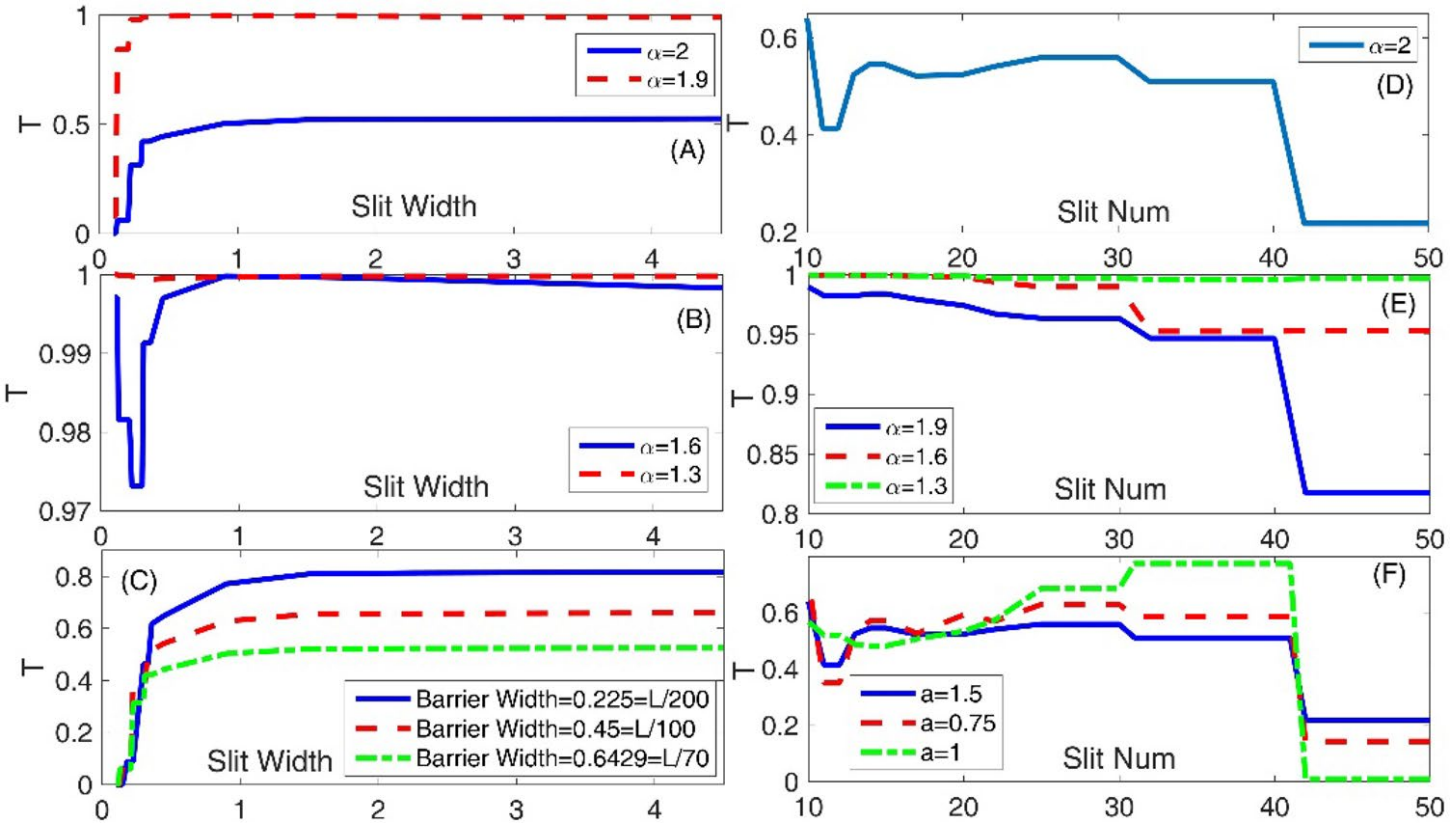
Panel (**A**) Variation of the transmission coefficient T as a function of the slit width for Schrodinger equations with $\alpha = 2$ and 1.9. Panel (**B**) The same as the panel (**A**) but for three fractional orders *α* = 1.6and 1.3. Panel (**C**) Variation of the transmission coefficient T as a function of the slit width for Schrodinger equations with $\alpha = 2$. Here the effects of three different values of the barrier widths are compared. Panel (**D**) Variation of the transmission coefficient T as a function of the number of slits for Schrodinger equations with $\alpha = 2$. Panel (**E**) The same as the panel (**D**) but for three fractional orders *α* = 1.9, 1.6 and 1.3. We assumed the layer width = L/30. Panel (**F**) Variation of the transmission coefficient T as a function of the number of slits for Schrodinger equations with $\alpha = 2$. Here the effects of three different values of the Gaussian wave parameter ‘a’ are compared. In this figure, we assumed system length L = 45, Laplacian coefficient *β* = 0.5, and nonlinearity strength *γ* = 0.

Now, in the panel (A) of the Fig. 5 we have plotted the variation of the transmission coefficient T as a function of the slit width for Schrodinger equations with $\alpha = 2$ and 1.9. Panel (B) is also the same as the panel (A) but for three fractional orders, *α* = 1.6 and 1.3. Besides, in the panel (C), we also observe that increasing the barrier width the transmission coefficient decreases. Panel (C) of this figure, shows the variation of the transmission coefficient T as a function of the slit width for Schrodinger equations with $\alpha = 2$. Here the effects of three different values of the barrier widths are compared. In the panels of this figure, we assumed system length L = 45, Laplacian coefficient $\beta = 0.5$, and nonlinearity strength $\gamma = 0$. As the panels (A–C) show, by increasing the slit width, the transmission coefficient monotonically increases. Another interesting fact (an exception) is also the system with the fractional parameter $\alpha = 1.6$ in the panel (B), within which as the slit width increases, the transmission coefficient at first decreases and then increases. This is a nonlinear behavior, which, again is not consistent with our conventional physical insight. At the time, we do not know its reason. Also, for slit widths larger than 1, the transmission coefficient, roughly speaking, does not depend on the slit width variations. This fact is also true for all studied fractional parameters and the barrier widths. An interesting point in these panels is the quantization of the transmission coefficient for systems with small slit. The quantization of the transmission is an intrinsic effect that does not change by a fractional degree or barrier width changes. In the meantime, panel (D) presents the variation of the transmission coefficient T as a function of the number of slits for Schrodinger equations with $\alpha = 2$. Also, panel (E) is the same as the panel (D) but for three fractional orders *α* = 1.9, 1.6 and 1.3. We assumed the layer width = L/30. Finally, the panel (F) illustrates the variation of the transmission coefficient T as a function of the number of slits for Schrodinger equations with $\alpha = 2$. Here the effects of three different values of the Gaussian wave parameter ‘a’ are compared. As the panels (D) and (E) show, as the fractional parameter $\alpha$ increases, the number of slits become less critical. Because, the variation of the slit number can make smaller changes in the transmission coefficient, and thus, the variation interval of the transmission coefficient becomes smaller. However, in the panel (F), we have not a common statement for variation of the transmission coefficient when the Gaussian wave parameter ‘a’ increases. In some slit number intervals the transmission coefficient increases if the Gaussian wave parameter ‘a’ increases and in some other intervals, the transmission coefficient decreases if the Gaussian wave parameter ‘a’ increases. But, there is a common fact in the panels (D–F). In these panels, we have some flat transmission diagrams within which the transmission coefficient does not change when the number of slits increases. This means we have different choices for the number of slits to have a typical transmission coefficient. Also, it means that in these slit number intervals, the transmission coefficient is quantized concerning the number of slits variations, and this is true for different values of the Gaussian wave parameter ‘a’ and fractional parameter $\alpha$.

**Figure 6:**
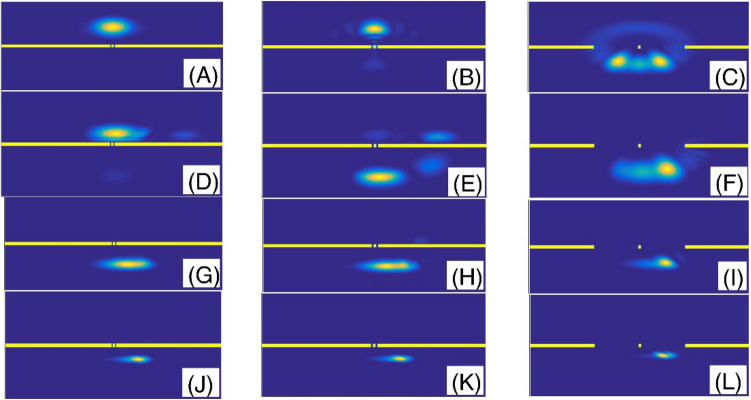
The final shape of the Gaussian wavepacket at sufficiently large time in the panels (**A**–**L**) are plotted for (slit width,*α*) = (L/450, 2), (L/200, 2), (L/10, 2), (L/450, 1.9), (L/200, 1.9), (L/10, 1.9), (L/450, 1.6), (L/200, 1.6), (L/10, 1.6), (L/450, 1.3), (L/200, 1.3), and (L/10, 1.3), respectively. Other parameters are the same as the panels (**A**) of the Fig. 5. In each panel, the wavepacket has moved downward.

**Figure 7:**
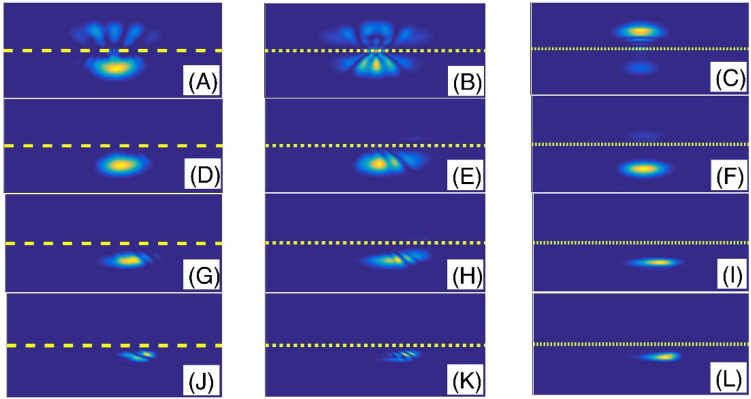
The final shape of the Gaussian wavepacket at sufficiently large time in the panels (**A**–**L**) are plotted for (number of slits,*α*) = (10, 2), (30, 2), (50, 2), (10, 1.9), (30, 1.9), (50, 1.9), (10, 1.6), (30, 1.6), (50, 1.6), (10, 1.3), (30, 1.3), and (50, 1.3), respectively. Other parameters are the as the panels (**A**) of the Fig. 5. In each panel, the wavepacket has moved downward.

Figure 6 shows the final shape of the Gaussian wavepacket at a sufficiently large time. The panels (A–L) are plotted for (slit width, $\alpha$) = (L/450, 2), (L/200, 2), (L/10, 2), (L/450, 1.9), (L/200, 1.9), (L/10, 1.9), (L/450, 1.6), (L/200, 1.6), (L/10, 1.6), (L/450, 1.3), (L/200, 1.3), and (L/10, 1.3), respectively. Other parameters are the same as the panels (A) of the Fig. 5. In each panel, the wavepacket has moved downward. These final states show the probability density distributions and illustrate the position of the fringes. Comparing the panels (J), (K), and (L) of the Fig. 6 shows that, in strongly fractional systems with the fractional parameter $\alpha = 1.3$, the wavepacket can surprisingly transmit through small and extensive slit width systems with approximately the same probability. Also, Fig. 7 shows the final shape of the Gaussian wavepacket at a sufficiently large time. The panels (A) to (L) are plotted for (number of slits, $\alpha$) = (10, 2), (30, 2), (50, 2), (10, 1.9), (30, 1.9), (50, 1.9), (10, 1.6), (30, 1.6), (50, 1.6), (10, 1.3), (30, 1.3), and (50, 1.3), respectively. Other parameters are the same as the panels (A) of the Fig. 5. In each panel, the wavepacket has moved downward. The main phenomenon in these panels is the wavepacket reconstruction after transmission through the multi-slit systems. However, this reconstruction is more perfect in the systems with the larger number of slits.

**Figure 8:**
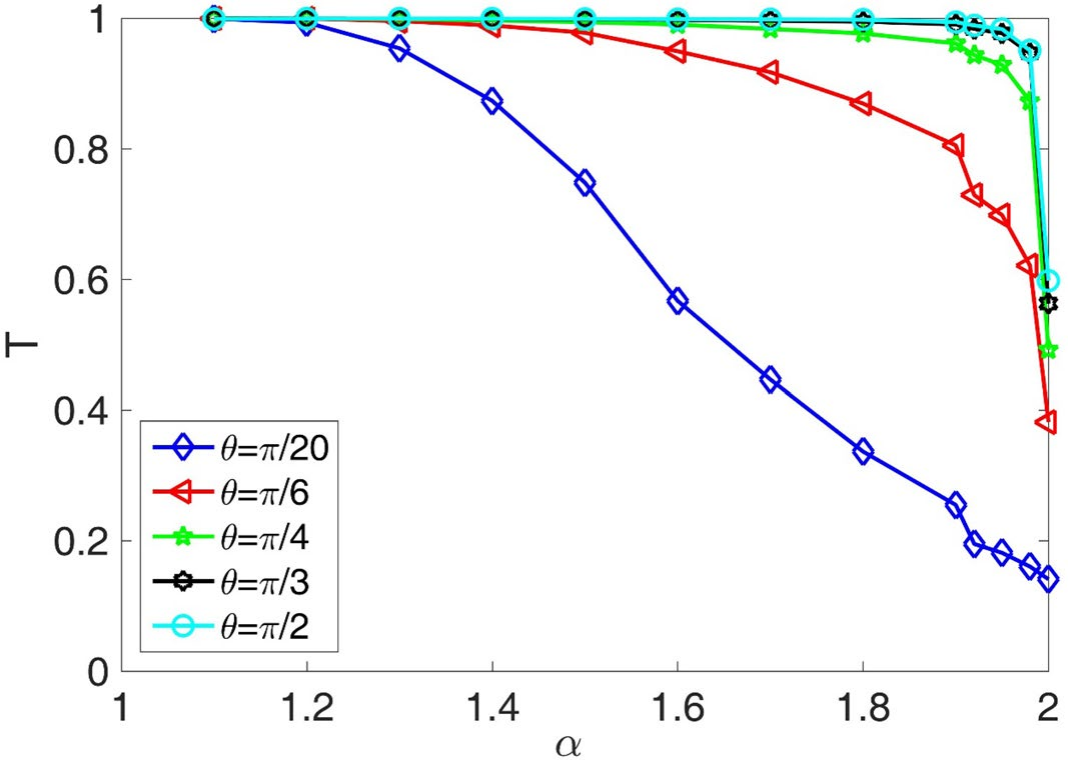
Variation of the transmission coefficient T as a function of the fractional orders *α*in fractional Schrodinger equation. The effects of five different incident angles are compared. In this figure, we assumed system length L = 100, initial Gaussian wave parameter a = 1.5, number of slits = 20, slit width = L/450, barrier width = L/70, layer width = L/150, layer height = 100, Laplacian coefficient *β* = 0.5, and nonlinearity strength *γ* = 0.

**Figure 9:**
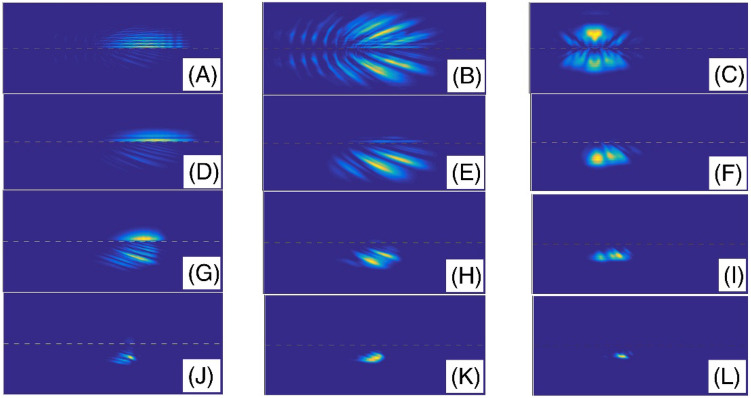
The final shape of the Gaussian wavepacket at sufficiently large time in the panels (**A**–**L**) are plotted for ($\theta$, *α*) = (pi/20, 2), (pi/4, 2), (pi/2, 2), (pi/20, 1.9), (pi/4, 1.9), (pi/2, 1.9), (pi/20, 1.6), (pi/4, 1.6), (pi/2, 1.6), (pi/20, 1.3), (pi/4, 1.3), and (pi/2, 1.3), respectively. Other parameters are the same as the panels (**A**) of the Fig. 8. In each panel, the wavepacket has moved downward.

In the following, the variation of the transmission coefficient T as a function of the fractional orders $\alpha$ in the fractional Schrodinger equation is presented in the Fig. 8. The effects of five different incident angles (with respect to the x-axis in the Fig. 1) are compared. In this figure, we assumed system length L = 100, initial Gaussian wave parameter a = 1.5, number of slits = 20, slit width = L/450, barrier width = L/70, layer width = L/150, layer height = 100, Laplacian coefficient $\beta = 0.{5}$, and nonlinearity strength $\gamma = 0$. As this figure shows, by increasing the fractional parameter, the transmission coefficient decreases. We see that, for the incident angels greater than π/4, the transmission coefficients are almost perfect for a large portion of the studied fractional parameters, and the transmission coefficient suddenly decreases at fractional parameters very close to $\alpha = 2$. However, for the incident angels smaller than π/4, the transmission coefficients can have values in the interval (0, 1) at all fractional parameter values. In Fig. 9, the final shape of the Gaussian wavepacket at a sufficiently large time for different values of the $\theta$ and $\alpha$ have also been illustrated.

**Figure 10:**
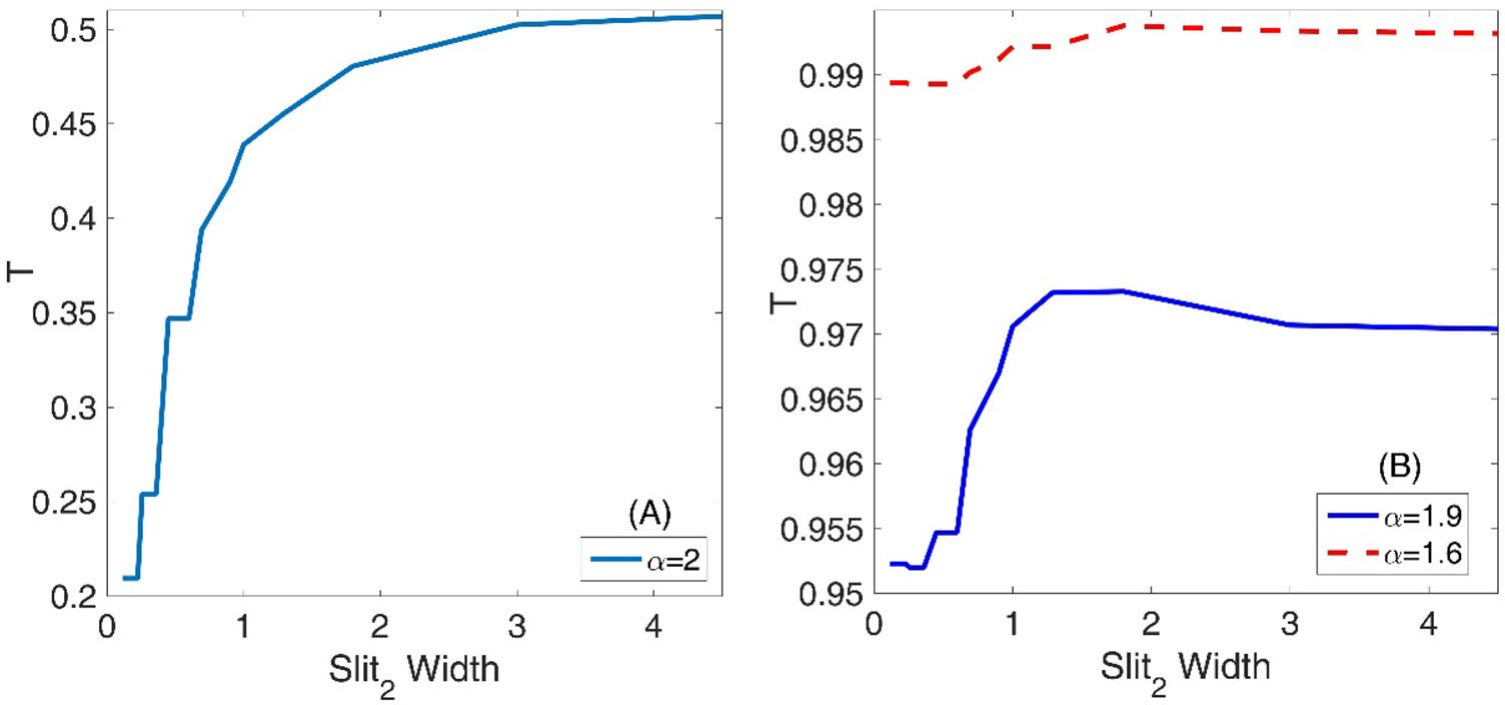
Panel (**A**) Variation of the transmission coefficient T as a function of the second slit width for standard non-fractional Schrodinger equation with $\alpha = 2$. Panel (**B**) The same as the panel (**A**) but for three fractional orders *α* = 1.9 and 1.6. In this figure, we assumed system length L = 45, initial Gaussian wave parameter a = 1.5, number of slits = 2, slit width = L/50, barrier width = L/70, layer width = L/30, layer height = 100, Laplacian coefficient *β* = 0.5, and nonlinearity strength *γ* = 0.

**Figure 11:**
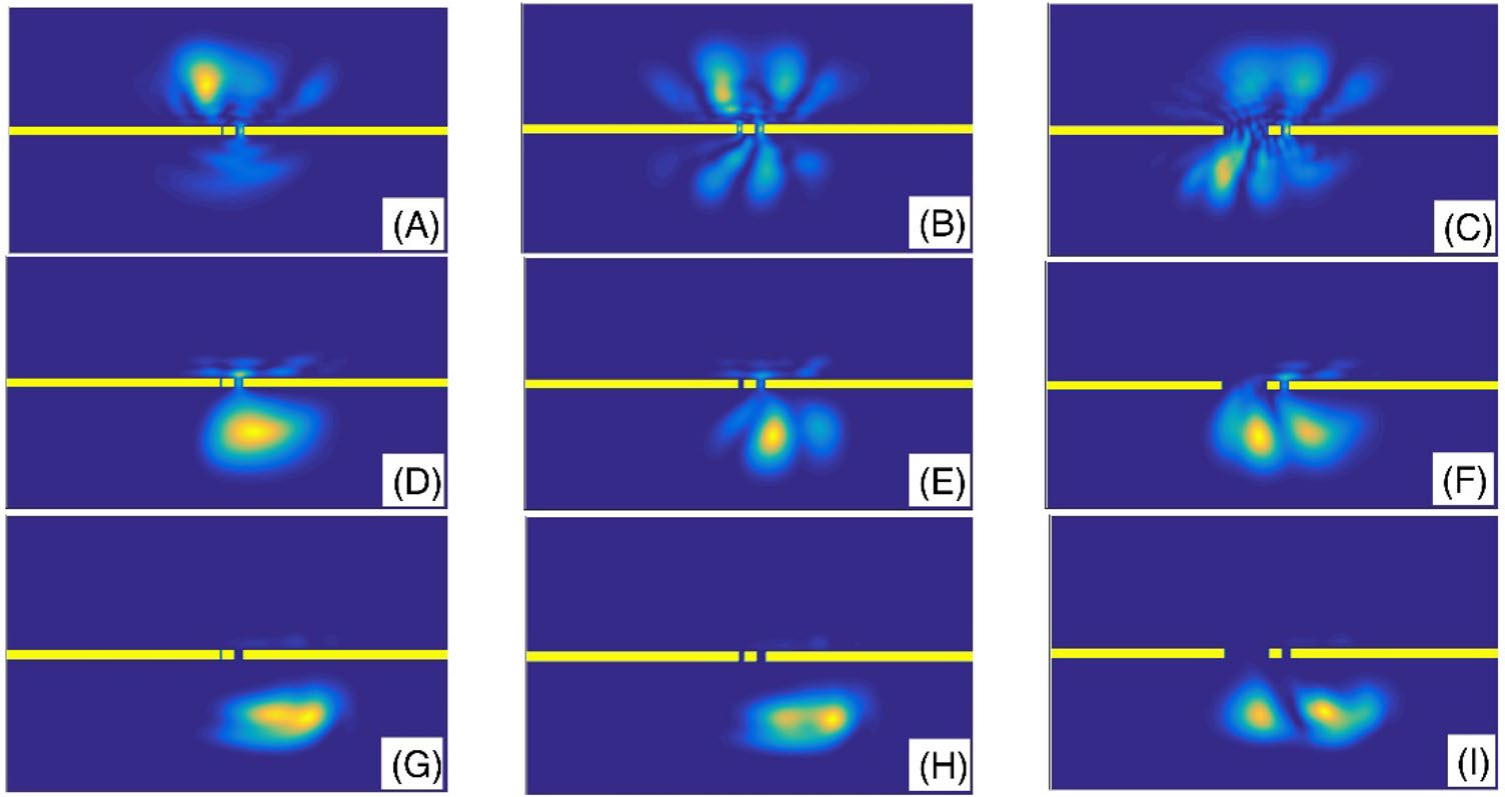
The final shape of the Gaussian wavepacket at a sufficiently large time in the panels (**A**–**I**) are plotted for (the second slit width,*α*) = (L/400, 2), (L/100, 2), (L/10, 2), (L/400, 1.9), (L/100, 1.9), (L/10, 1.9), (L/400, 1.6), (L/100, 1.6), and (L/10, 1.6), respectively. Other parameters are the same as the panels (**A**) of the Fig. 10. In each panel, the wavepacket has moved downward.

We present the variation of the transmission coefficient T as a function of the second slit width for standard non-fractional Schrodinger equation with $\alpha = 2$ in the panel (A) of the Fig. 10. Therefore, we study a double-slit that the sizes of the slits are not equal. Panel (B) also is the same as the panel (A) but for three fractional orders *α* = 1.9 and 1.6. In this figure, we assumed system length L = 45, initial Gaussian wave parameter a = 1.5, number of slits = 2, slit width = L/50, barrier width = L/70, layer width = L/30, layer height = 100, Laplacian coefficient $\beta = 0.5$, and nonlinearity strength $\gamma = 0$. Here also, for small values of the slit width and for fractional orders, *α* = 1.9 and 2, the transmission coefficient is quantized concerning it. An extraordinary fact in the panel (B) is also that the diagram of the transmission coefficient as a function of the slit width in a nonlinear one. Besides, for large values of the slit width that are greater than 3, all three diagrams saturate, and further increasing the slit width does not change the transmission coefficient any more. Also, the Fig. 11 shows the final shape of the Gaussian wavepacket at a sufficiently large time for different values of the second slit width and $\alpha$. In this figure, we see that, in some situations such as in the panel (D), the wave function reconstruction can occur after the transmission of the wavepacket through the double-slit system. Considering the panels (G–I) also reveals that the wavepacket can transmit the systems with different small and large slit widths by approximately the same probability. According to the Fig. 10, these probabilities are in the interval (0.99, 1).

**Figure 12:**
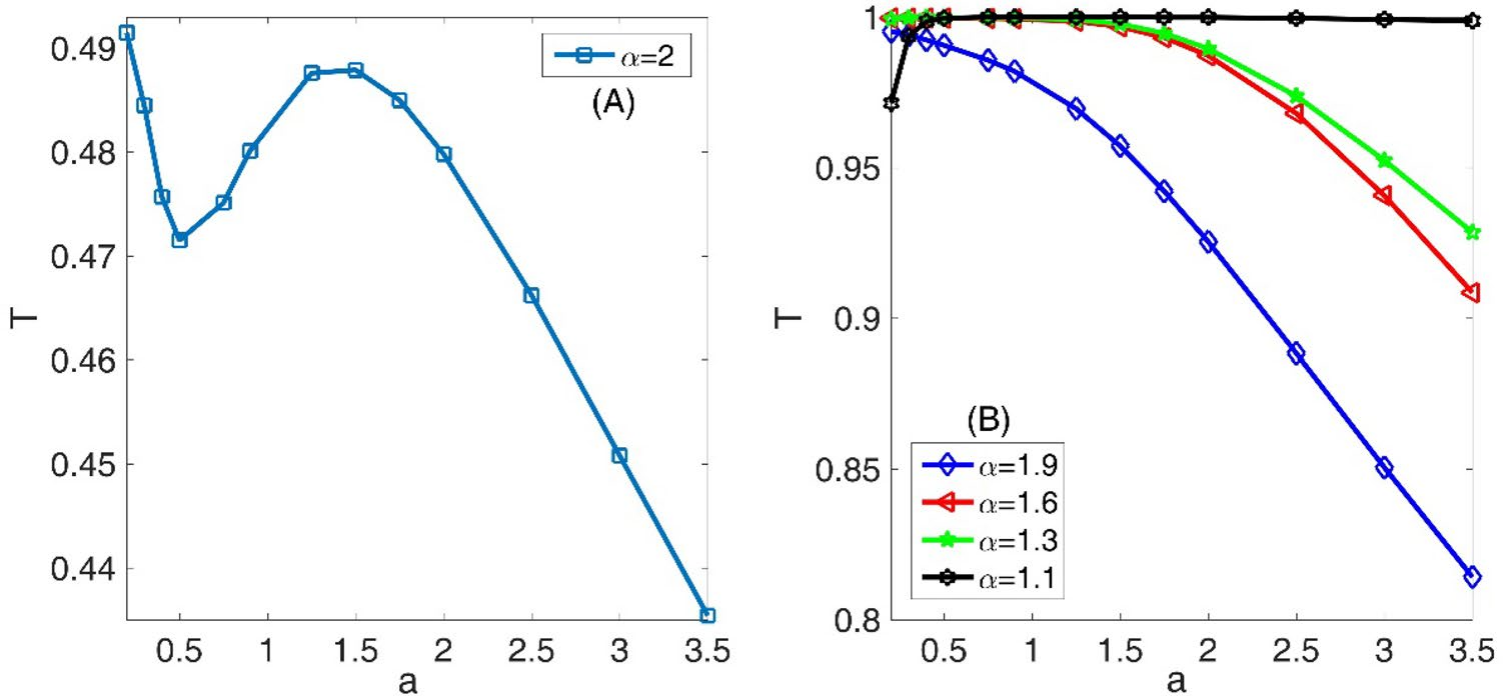
Panel (**A**) Variation of the transmission coefficient T as a function of Gaussian wave parameter a for standard non-fractional Schrodinger equation with $\alpha = 2$. Panel (**B**) The same as the panel (**A**) but for three fractional orders *α* = 1.9, 1.6, 1.3, and 1.1. In this figure, we assumed system length L = 45, number of slits = 2, slit width = L/50, barrier width = L/70, layer width = L/30, layer height = 100, Laplacian coefficient *β* = 0.5, and nonlinearity strength *γ* = 0.

**Figure 13:**
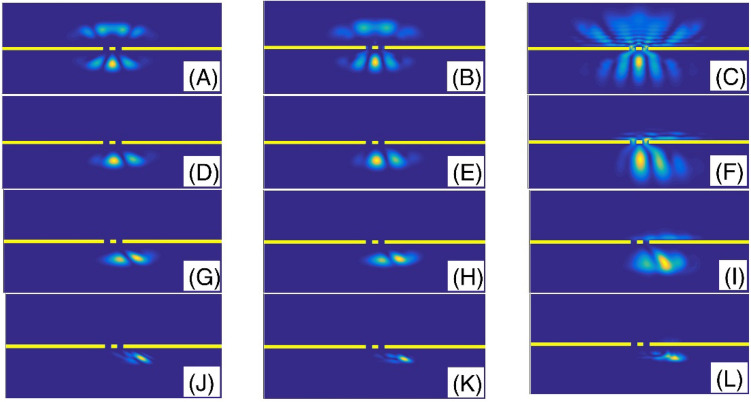
The final shape of the Gaussian wavepacket at sufficiently large time in the panels (**A**–**L**) are plotted for (a,*α*) = (0.2, 2), (0.5, 2), (3.5, 2), (0.2, 1.9), (0.5, 1.9), (3.5, 1.9), (0.2, 1.6), (0.5, 1.6), (3.5, 1.6), (0.2, 1.3), (0.5, 1.3), and (3.5, 1.3), respectively. Other parameters are the same as the panels (**A**) of the Fig. 12. In each panel the wavepacket has moved downward.

In the panel (A) of the Fig. 12, we have presented the variation of the transmission coefficient T as a function of Gaussian wave parameter ‘a’ for standard non-fractional Schrodinger equation with $\alpha = 2$. Panel (B) is also the same as the panel (A) but for three fractional orders *α* = 1.9, 1.6, 1.3 and 1.1. In this figure, we assumed system length L = 45, number of slits = 2, slit width = L/50, barrier width = L/70, layer width = L/30, layer height = 100, Laplacian coefficient *β* = 0.5, and nonlinearity strength *γ* = 0. Comparing the panels (A) and (B) shows that the behavior of the wavepacket in the standard Schrodinger equation is completely different from propagation characteristics in the fractional system. In the panel (A), there are two characteristic wavepacket widths for them that the diagram of the transmission coefficient has a local minimum and a local maximum. The interesting region is the interval between these two characteristic wavepacket widths. In this region, by increasing the wavepacket widths, the transmission coefficient increases that is not consistent with our common physics insight. In the panel (B), there is a similar behavior for the system with fractional orders *α* = 1.9 for wavepacket width smaller than 0.25. Another interesting fact is that for wavepacket width larger than 0.25, the transmission coefficient is 100% for the systems with the fractional orders *α* = 1.1. Figure 13 also shows the final shape of the Gaussian wavepacket at a sufficiently large time for different values of the Gaussian wave parameter ‘a’. A result in this figure is that the wave function is distributed in a larger spatial region for the wavepackets with larger widths. Besides, by decreasing the fractional parameter $\alpha$, the wavepacket is localized within a smaller region.

**Figure 14:**
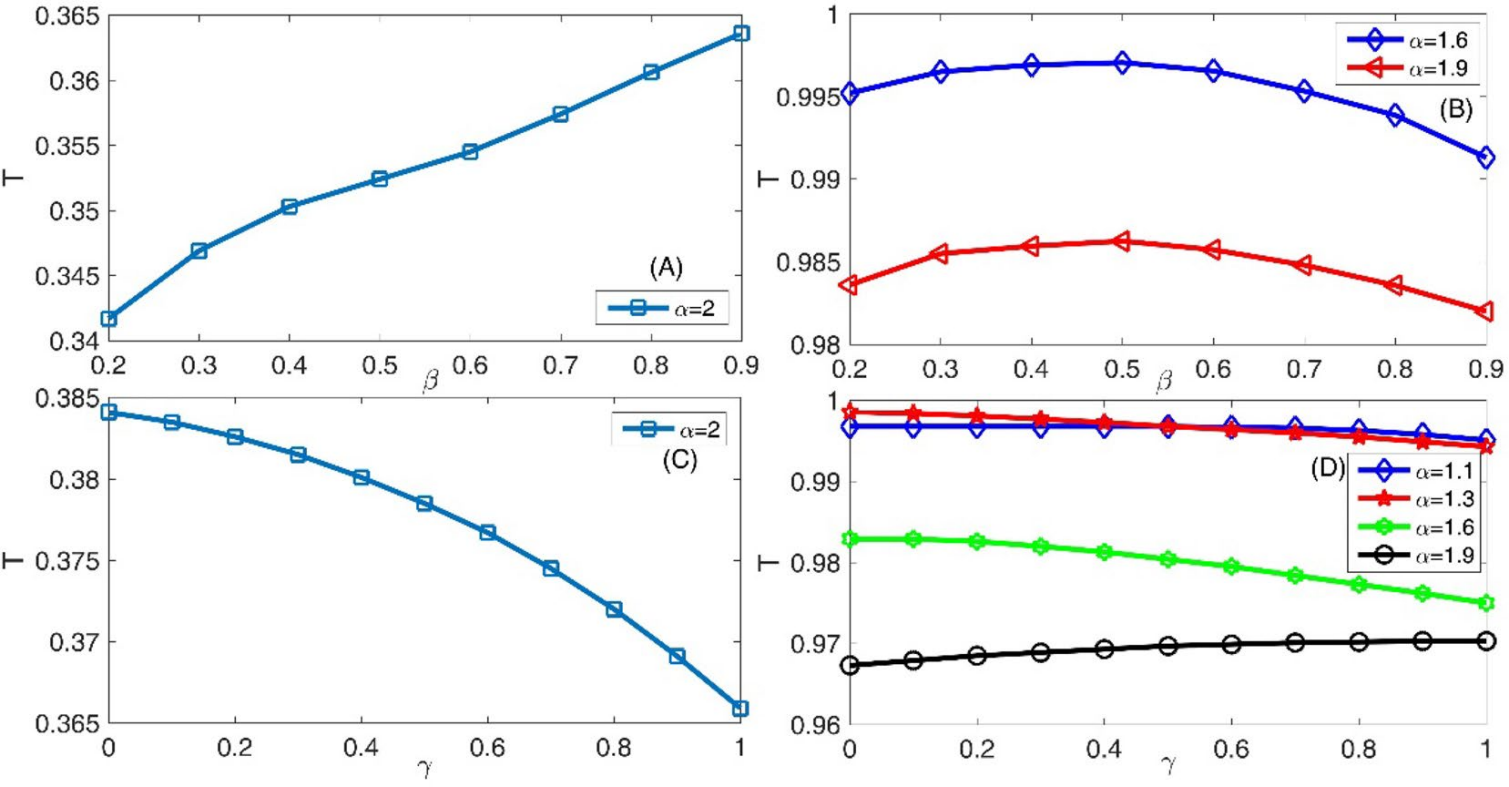
Panel (**A**) Variation of the transmission coefficient T as a function of the Laplacian coefficient *β* for standard non-fractional Schrodinger equation with $\alpha = 2$. Panel (**B**) The same as the panel (**A**) but for three fractional orders *α* = 1.9 and 1.6. Panel (**C**) Variation of the transmission coefficient T as a function of nonlinearity strength *γ* for standard non-fractional Schrodinger equation with $\alpha = 2$. Panel (**D**) The same as the panel (**C**) but for three fractional orders *α* = 1.9, 1.6, 1.3 and 1.1. In this figure, we assumed system length L = 45, number of slits = 2, slit width = L/100, barrier width = L/60, layer width = L/30, and layer height = 100.

**Figure 15:**
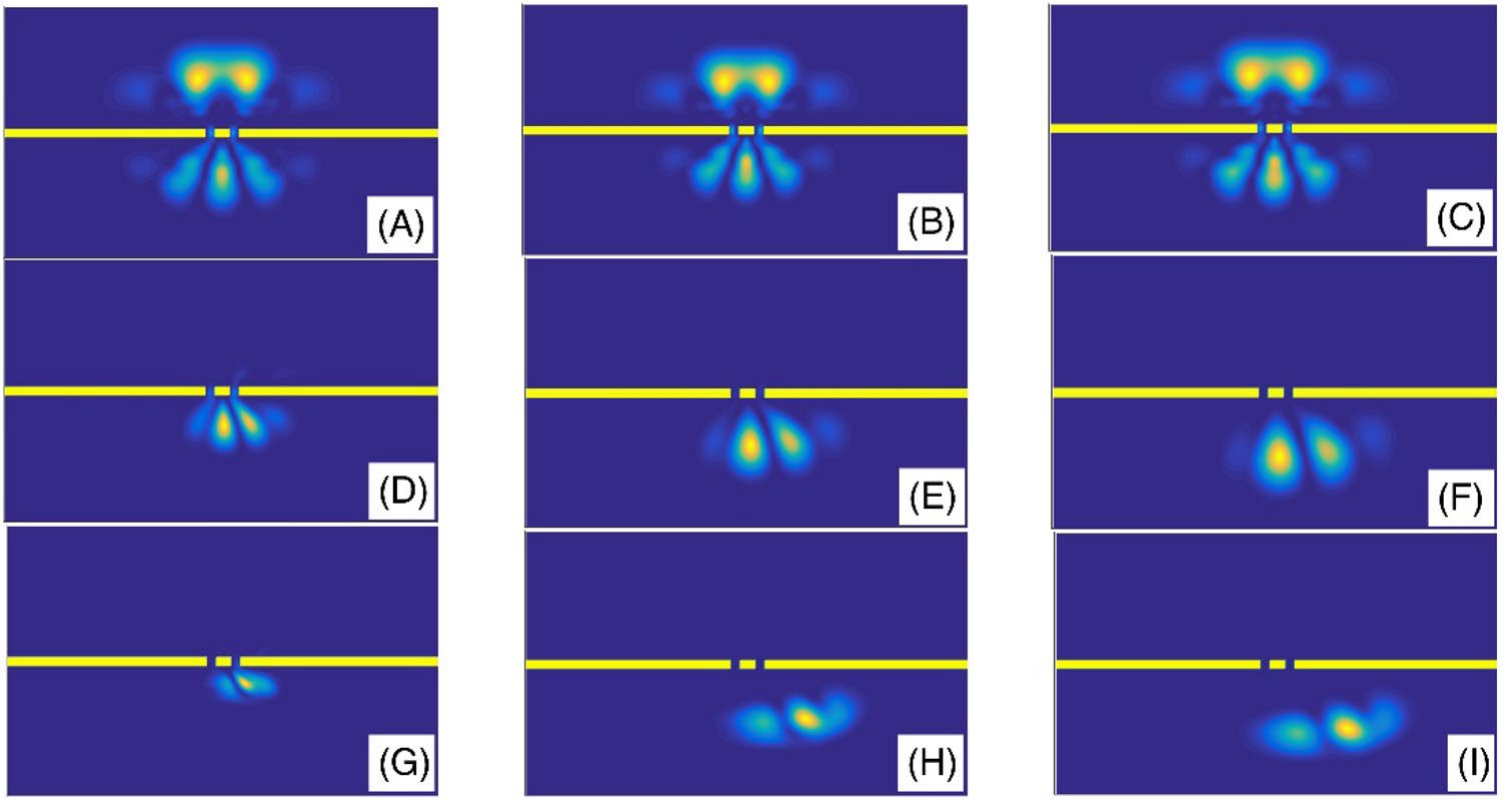
The final shape of the Gaussian wavepacket at sufficiently large time in the panels (**A**–**I**) are plotted for (*β*, *α*) = (0.2, 2), (0.5, 2), (0.9, 2), (0.2, 1.9), (0.5, 1.9), (0.9, 1.9), (0.2, 1.6), (0.5, 1.6), and (0.9, 1.6), respectively. Other parameters are the same as the panels (**A**) of the Fig. 14. In each panel, the wavepacket has moved downward.

**Figure 16:**
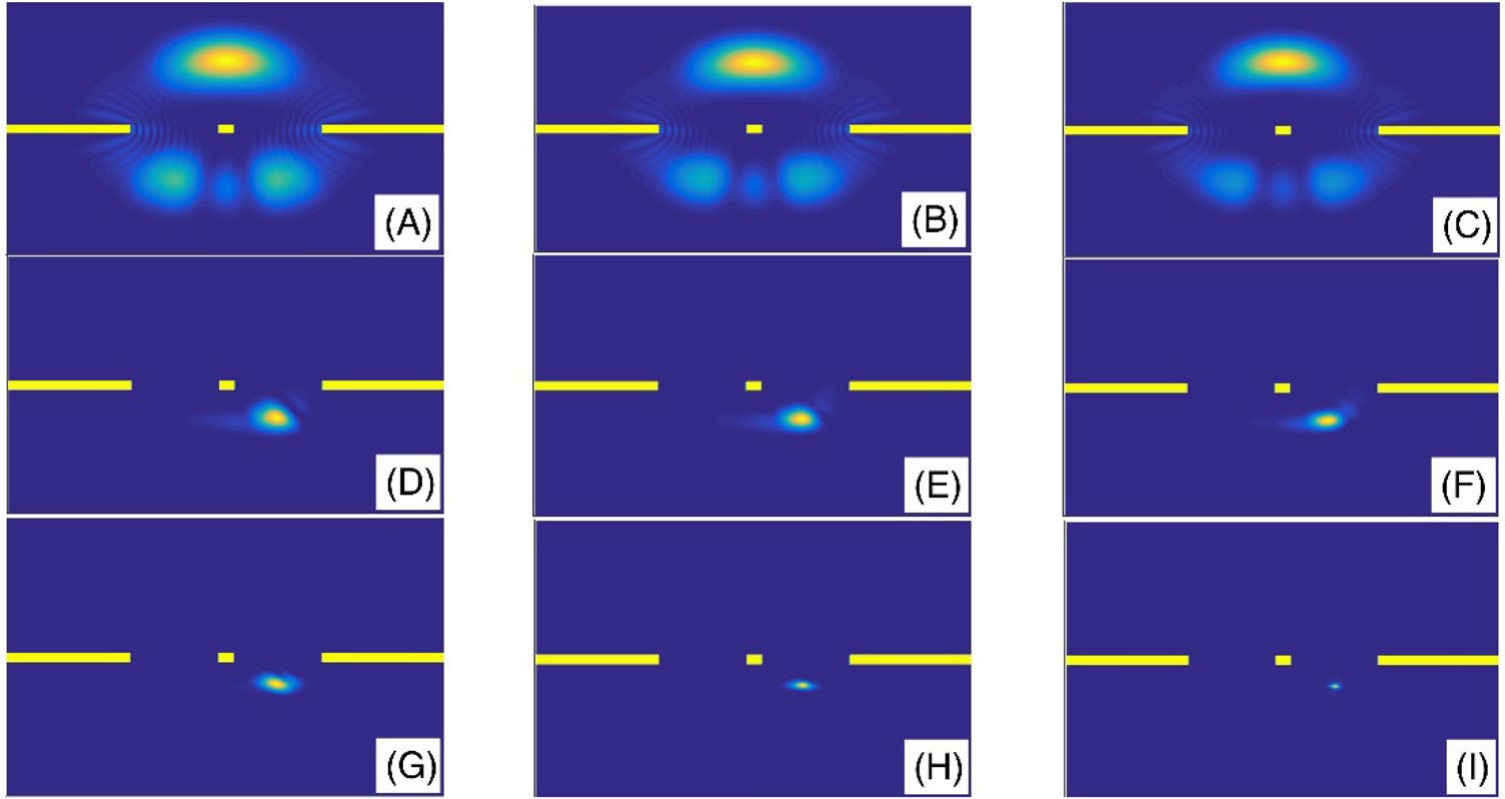
The final shape of the Gaussian wavepacket at sufficiently large time in the panels (**A**–**I**) are plotted for (*γ*, *α*) = (0, 2), (0.5, 2), (1, 2), (0, 1.6), (0.5, 1.6), (1, 1.6), (0, 1.3), (0.5, 1.3), and (1, 1.3), respectively. Other parameters are the same as the panels (**A**) of the Fig. 14. In each panel, the wavepacket has moved downward.

Finally, in panel (A) of the Fig. 14, we have presented the variation of the transmission coefficient T as a function of the Laplacian coefficient $\beta$ for the standard non-fractional Schrodinger equation with $\alpha = 2$. Panel (B) also illustrates the same as the panel (A) but for three fractional orders, *α* = 1.9 and 1.6. As we mentioned in the introduction section, the fractional Laplacian means a non-parabolic dispersion, i.e., the dispersion of the system directly changes. In the standard Schrodinger equation on the panel (A), the transmission coefficient varies monotonically in an increasing manner, when the Laplacian coefficient increases. However, in the studied diagrams in the panel (B), the transmission diagrams have a maximum value. This means there is a critical Laplacian coefficient that leads to the maximum transmission coefficient. Figure 15 illustrates the final shape of the Gaussian wavepacket at a sufficiently large time for different values of the Laplacian coefficient $\beta$. Other parameters are the same as the panels (A) of the Fig. 14. In each panel the wavepacket has moved downward. In these panels, we can see the wavepacket localization and its evolution. In the panel (C) we have shown the variation of the transmission coefficient T as a function of nonlinearity strength *γ* for standard non-fractional Schrodinger equation with $\alpha = 2$. Also, panel (D) is the same as the panel (C) but for three fractional orders *α* = 1.9, 1.6, 1.3 and 1.1. In this figure, we assumed system length L = 45, number of slits = 2, slit width = L/100, barrier width = L/60, layer width = L/30, and layer height = 100. When the nonlinearity strength *γ* increases, the transmission coefficient decreases. However, the fractional order *α* = 1.9 is an exception, and in this case, we have an increasing diagram. Finally, Fig. 16, presents the final shape of the Gaussian wavepacket at a sufficiently large time in the panels for different values of the nonlinearity strength *γ*.Other parameters are the same as the panels (A) of the Fig. 14. In each panel, the wavepacket has moved downward.

## Conclusion

In the current work, we studied the transmission through double and multi-slits systems as well as wavepacket evolution in the fractional Schrodinger framework. We showed that, by increasing the layer height, the transmission coefficient decreases in the standard Schrodinger equation, but in strongly fractional Schrodinger equation, the transmission coefficient at first decreases and then increases if layer height increases. By decreasing the fractional parameter *α* the transmission coefficient increased. For systems with small slit, the transmission coefficient was quantized when the slit width changed. By increasing the barrier width the transmission coefficient decreased. In some slit number intervals, the transmission coefficient was quantized concerning the number of slits variations and this was true for different values of the Gaussian wave parameter ‘a’ and fractional parameter *α*. In strongly fractional systems, the wavepacket could transmit through small and extensive slit width systems with approximately the same probability. We observed the wavepacket reconstruction after transmission through the multi-slit systems and this reconstruction was more perfect in the systems with a larger number of slits. For the incident angels greater that π/4, the transmission coefficients were almost perfect for many studied fractional parameters. For some values of the wavepacket widths, by increasing the wavepacket widths, the transmission coefficient increased. By decreasing the fractional parameter *α*, the wavepacket was localized within a smaller region. Finally, the double slit interference pattern exists for fractional cases, but by increasing the degree of fractionality (decreasing the fractional order α), the interference effect gradually vanishes.

## Supplementary information

Supplementary video legends.

Supplementary video 1-1-1.

Supplementary video 1-1-2.

Supplementary video 1-1-3.

Supplementary video 1-1-4.

Supplementary video 1-1-5.

Supplementary video 1-1-6.

Supplementary video 1-1-7.

Supplementary video 1-1-8.

Supplementary video 1-1-9.

Supplementary video 1-1-10.

Supplementary video 1-1-11.

Supplementary video 1-1-12.

Supplementary video 1-2-1.

Supplementary video 1-2-2.

Supplementary video 1-2-3.

Supplementary video 1-2-4.

Supplementary video 1-2-5.

Supplementary video 1-2-6.

Supplementary video 1-2-7.

Supplementary video 1-2-8.

Supplementary video 1-2-9.

Supplementary video 1-2-10.

Supplementary video 1-2-11.

Supplementary video 1-2-12.

Supplementary video 2-1-1.

Supplementary video 2-1-2.

Supplementary video 2-1-3.

Supplementary video 2-1-4.

Supplementary video 2-1-5.

Supplementary video 2-1-6.

Supplementary video 2-1-7.

Supplementary video 2-1-8.

Supplementary video 2-1-9.

Supplementary video 2-1-10.

Supplementary video 2-1-11.

Supplementary video 2-1-12.

Supplementary video 2-2-1.

Supplementary video 2-2-2.

Supplementary video 2-2-3.

Supplementary video 2-2-4.

Supplementary video 2-2-5.

Supplementary video 2-2-6.

Supplementary video 2-2-7.

Supplementary video 2-2-8.

Supplementary video 2-2-9.

Supplementary video 2-2-10.

Supplementary video 2-2-11.

Supplementary video 2-2-12.

Supplementary video 3-1.

Supplementary video 3-2.

Supplementary video 3-3.

Supplementary video 3-4.

Supplementary video 3-5.

Supplementary video 3-6.

Supplementary video 3-7.

Supplementary video 3-8.

Supplementary video 3-9.

Supplementary video 3-10.

Supplementary video 3-11.

Supplementary video 3-12.

Supplementary video 4-1.

Supplementary video 4-2.

Supplementary video 4-3.

Supplementary video 4-4.

Supplementary video 4-5.

Supplementary video 4-6.

Supplementary video 4-7.

Supplementary video 4-8.

Supplementary video 4-9.

Supplementary video 5-1.

Supplementary video 5-2.

Supplementary video 5-3.

Supplementary video 5-4.

Supplementary video 5-5.

Supplementary video 5-6.

Supplementary video 5-7.

Supplementary video 5-8.

Supplementary video 5-9.

Supplementary video 5-10.

Supplementary video 5-11.

Supplementary video 5-12.

Supplementary video 6-1-1.

Supplementary video 6-1-2.

Supplementary video 6-1-3.

Supplementary video 6-1-4.

Supplementary video 6-1-5.

Supplementary video 6-1-6.

Supplementary video 6-1-7.

Supplementary video 6-1-8.

Supplementary video 6-1-9.

Supplementary video 6-2-1.

Supplementary video 6-2-2.

Supplementary video 6-2-3.

Supplementary video 6-2-4.

Supplementary video 6-2-5.

Supplementary video 6-2-6.

Supplementary video 6-2-7.

Supplementary video 6-2-8.

Supplementary video 6-2-9.
