# Author Correction: Fractional Young double-slit numerical experiment with Gaussian wavepackets

**DOI:** 10.1038/s41598-021-88754-y

**Published:** 2021-05-06

**Authors:** Mahboubeh Ghalandari, M. Solaimani

**Affiliations:** Department of Physics, Qom University of Technology, Qom, Iran

Correction to: *Scientific Reports* 10.1038/s41598-020-76512-5, published online 10 November 2020

The original version of this Article contained errors in the Formalism section, where Equation 1 and the subsequent formula were incorrect and Equations 2–4 were omitted.

“The wavepacket propagation in the two-dimensional fractional Schrodinger equation formalism can be studied by using:

1 $$i\frac{\partial }{\partial t}\psi \left( {x,y,t} \right) = \left[ {{\beta }\left( { - \Delta} \right)^{\alpha /2} - \gamma \left| {\psi \left( {x,y,t} \right)} \right|^{2} + V\left( {x,y} \right)} \right]\psi \left( {x,y,t} \right)$$

where ${\alpha }$, $\beta$, and ${\gamma }$ are the fractional derivative order, Laplacian coefficient, and nonlinear interaction strength, respectively. We also have $\Delta = \partial^{2} /\partial x^{2} + \partial^{2} /\partial y^{2}$.”

now reads:

“The wavepacket propagation in the two-dimensional fractional Schrodinger equation formalism can be studied by using:

1 $$i\frac{\partial \psi }{{\partial t}} = \left[ {{\upbeta }{\text{Q}}_{{\text{R}}} \left( {{\text{x}},{\text{y}},t,\alpha } \right)\left( { - \Delta^{2} } \right)^{{\frac{\alpha }{2}}} - \gamma \left| {\psi \left( {x,y,t} \right)} \right|^{2} + {\text{M}}\left( {{\text{x}},{\text{y}},p_{x} ,p_{y} ,t,\alpha } \right)} \right]\psi \left( {x,y,t} \right)$$

where ${\alpha }$, ${\upbeta}$, and ${\gamma }$ are the fractional derivative order, Laplacian coefficient, and nonlinear interaction strength, respectively. We also have $\Delta^{2} = \partial^{2} /\partial x^{2} + \partial^{2} /\partial y^{2}$. Also ${\text{Q}}_{{\text{R}}}$, and M are real and complex functions, respectively. Let us assume that

2 $${\text{M}}\left( {{\text{x}},{\text{y}},p_{x} ,p_{y} ,t,\alpha } \right) = {\text{V}}\left( {x,y} \right) + i{\upbeta }{\text{Q}}_{{\text{I}}} \left( {{\text{x}},{\text{y}},t,\alpha } \right)\left( { - \Delta^{2} } \right)^{{\frac{\alpha }{2}}}$$

where the geometrical potential ${\text{V}}\left( {x,y} \right)$ is defined for the double slit problem, and ${\upbeta }{\text{Q}}_{{\text{I}}} \left( {{\text{x}},{\text{y}},t,\alpha } \right)$ is a real function that determines amplitude of imaginary part of the potential. By substituting Eq. (2) in Eq. (1), we have

3 $$i\frac{\partial \psi }{{\partial t}} = \left[ {{\upbeta }{\text{Q}}\left( {{\text{x}},{\text{y}},t,\alpha } \right)\left( { - \Delta^{2} } \right)^{{\frac{\alpha }{2}}} - \gamma \left| {\psi \left( {x,y,t} \right)} \right|^{2} + {\text{V}}\left( {{\text{x}},{\text{y}}} \right)} \right]\psi \left( {x,y,t} \right)$$

where $${\text{Q}}\left( {{\text{x}},{\text{y}},t,\alpha } \right) = {\text{Q}}_{R} \left( {{\text{x}},{\text{y}},t,\alpha } \right) + {\text{ iQ}}_{{\text{I}}} \left( {{\text{x}},{\text{y}},t,\alpha } \right)$$.

Note that when ${\text{Q}}\left( {{\text{x}},{\text{y}},t,\alpha } \right) = {\text{exp}}\left( {2\pi i} \right) = 1$, we obtain the usual NFSE. In this paper however, we investigated a more general case of ${\text{Q}}\left( {{\text{x}},{\text{y}},t,\alpha } \right)$ as follows

4 $${\text{Q}}\left( {{\text{x}},{\text{y}},t,\alpha } \right) = \exp \left( {\frac{i\pi \alpha }{{2\left| {g\left( {{\text{x}},{\text{y}},t} \right)} \right|}}\left( {\left| {g\left( {{\text{x}},{\text{y}},t} \right)} \right| - g\left( {{\text{x}},{\text{y}},t} \right)} \right)} \right)$$

where $g\left( {{\text{x}},{\text{y}},t} \right) = - i\frac{{\Delta \psi \left( {x,y,t} \right)}}{{\psi \left( {x,y,t} \right)}}$ is a real function.”

As a result, Equations 5–8 were originally listed as Equations 2–5.

The original Article has been corrected.

